# Orthopedic Surgery in Europe

**Published:** 1846-08

**Authors:** Buckminster Brown


3. *Orthopedic Surgery in Europe.*—Paris, May 19th, 1846. To the Editor of the Boston Medical and Surgical Journal.

DEAR SIR,—In Paris, during the last winter, I have found, as every medical man must find, numerous objects of deep interest; but my attention has been chiefly fixed upon subjects connected with orthopedy. Through the kindness of Messrs. Guerin, Bouvier and others, I have had many opportunities of observation in this most interesting branch of science. I have found in Guerin's wards, at the Hôpital des Enfans Malades, and at his weekly consultations, many cases of great interest. In addition to this, he has, at his own house, weekly meetings of medical gentlemen, for conversation on whatever matters of importance may have presented themselves during the week. Among the orthopedic cases at the Hospital, there were three or four in the various stages of treatment for congenital dislocation or luxation of the hip. To these I paid much attention, and watched their progress with care. Guerin's treatment and theory, in these cases, consists—Firstly, in continued extension of the limb. This is accomplished by means of a weight and pulley. There is a long, leather splint, well cushioned, applied to the leg, embracing it from the knee to the ankle. In this two rings are inserted—one for a cord which runs through a pulley at the foot of the couch, and to which is attached the weight. This is strictly and simply the course "preparatoire." By this means, in the course of a longer or shorter period, according to the nature of the case, the head of the femur gradually descends to a level with that of the sound limb. This all-important object being accomplished, the second stage of the treatment, or the process of "creusant," is commenced. A simple yet ingenious contrivance is used for this purpose; and the head of the femur in its new position is kept in almost constant action upon the acetabulum, thus excavating for itself a new socket, or rather, I should say, deepening that which we may suppose to have previously existed.

Guerin's theory in this respect is, that in no case is the cotyloid cavity entirely wanting. This he affirms as the result of his own observations on the numerous cases of congenital luxation which he has treated, but more particularly have the various *post-mortem* examinations, made either by himself or by his assistant, M. Kuhn (to whom, *post-mortem*

examinations of deformity is a subject of deep interest, and one to which he has devoted much time and attention,) confirmed him in his opinion. In every case a cavity has been found. In many cases, no doubt, it is extremely shallow, and in adults nearly obliterated, but never entirely. The patient who has been subjected to the mode of treatment of which I have been speaking, is often, after a certain length of time, permitted gradually to make use of more active exercise, and he proceeds from simply swinging the limb, to the walking stool, in which the chief part of the weight is taken from the still feeble member, and there, as the process of cure continues, he is enabled to make a free use of his own feet.

It may be, however, that before the first part of the process can be accomplished, namely, the descent of the head of the bone, the aid of tenotomy will be required. This may be termed the second class. In this case it may become necessary to divide the various muscles which by their contraction offer an opposition to our efforts. As, for example, I can recall a case in which the tendon of the two adductors, the gluteus medius and minimus, the psoas, the rectus femoris, as also (the case being complicated) the biceps, the external lateral ligament and the tendo-Achilles, were each in their turn divided. But in that class of cases, in which the depression is so extremely shallow as to render vain the attempt to secure the head of the bone in its normal position by this course of treatment, a mode more bold and active must be adopted. In these cases Guerin performs an operation, which first suggested itself to him, as the result of these more general applications of the fundamental principle upon which the operation by the subcutaneous method has taken its stand, and the more extended application of which he is the acknowledged originator. This operation resembles that which he performs for the radical cure of hernia. The head of the femur rests in all these cases on the dorsum of the ilium; the capsular ligament is of necessity much elongated, being stretched, according to the extent of the deformity, either one inch and a half, or two, or sometimes even three inches. The luxation having been reduced either by the simple means, or by division of the muscles, and the process of passive exercise, &c., having been tried without success, there being still, after a proper length of time has elapsed, a constant disposition of the bone to return to its abnormal position, slipping from its place when the slightest weight is applied to it, the operation then becomes necessary. Guerin introduces his instrument from without inward, and carries it down to the capsular ligament, which he cuts across upon a level with the upper lip of the socket. By this means effusion of coagulable lymph is produced. There is adhesion and cicatrization, with its necessary result, contraction. In fifteen days gentle passive motion is made use of, and in time a firm ligament is formed, by which the head of the bone is held securely in its new position. Immediately after the operation a band is placed firmly round the pelvis, with a compress upon the joint. Several other cases were described to me, besides those I have seen at the Hospital, some of double, some of single congenital luxation, in which the operation has been attended with the most favorable results.

4th. There is yet another class of cases. In certain children the resistance to the means employed for producing a descent of the head of the femur, is so great as to render these efforts wholly ineffectual. What is very curious in these cases is, that in lieu of this, there is an elongation of the bone itself, by which ample compensation is made. Thus we have still the signs of dislocation on examining the hip-joint, but on comparing the two limbs we find them of the same length. There was one very fine example of this in the Hospital, affording by accurate measurement, positive proof of the occurrence of this elongation. The patient, in this case, will have, of course, a slight awkwardness in his gait, but without the usual limp. I will not leave this subject without briefly remarking that in this, as, in truth, is the case in Paris, upon almost every subject connected with medicine, there has been much controversy, and that M. Guerin and Bouvier have arrayed themselves upon opposite sides. Of course I shall not attempt to form a decided opinion until I have had a still greater number of cases presented to my view, and have had that opportunity for careful and accurate observation which private practice can alone afford.

As a curious example of the truth of the above remark, and of the thorough investigation which all things here undergo, having any connection with the science of medicine, may be cited the controversy which has been going on the past winter between MM. Velpeau and Blandier on the treatment of hydrocele, with the particulars of which you are no doubt well acquainted.

The course of treatment pursued by Messieurs Guerin and Bouvier, and by Dr. Little, of London, for the various phases of spinal disease, is the same as that employed at the Boston Orthopedic Institution, with the exception that the above-named gentlemen adopt the prone position in a somewhat greater number of cases than has been the practice in Boston. The only reason why this should not be more generally made use of in certain cases, is the wearisomeness of the position, almost entirely debarring the patient from the amusements of which the other positions admit.

In some cases no doubt this mode of treatment is very important, as in scrofulous disease with excurvation, where the anterior portion of the bodies of some of the vertebræ are in a state of caries. Here the prone position is undoubtedly the one which affords the greatest promise of success. and should in all cases be employed where the patient can be prevailed upon to submit to it. To be used with the expectation of a favorable result, there is much minutiæ to be attended to in regard to the formation of the couch, adjustment of the cushions, together with the appropriate body apparatus so arranged that while the patient is recumbent there shall be a gentle elastic pressure constantly exerted upon the protuberant part. Guerin remarks that the course pursued should be the same as that for the fracture of a limb, and that, as far as this part of the treatment is concerned, this disease should be viewed in the same light.

It is greatly to be regretted that in Paris the provision for that large class of sufferers who are afflicted with some of the various deformities which recent advances in science have so well prepared the surgeon to relieve, but who have the additional misfortune of poverty, should be so inferior and so unworthy the results which might be effected under other circumstances, and which has been brought about in private practice. From the great error which was committed in the first instance of placing the patients of this class in one of their large public hospitals, merely allotting two or three of the smaller wards to their reception, it has seemed to me impossible that in certain cases the surgeon should be able to do either himself or his patients justice. This is more especially the case in lateral curvature, and general feebleness of the muscular and nervous systems, where the all-important auxiliaries of gymnastic exercises, suited to attain the peculiar object in view, be it the development of a particular set of muscles, or the general strengthening of all the muscles, or quickening the dormant circulation and giving vigor to the debilitated nerves, must be for the most part abandoned for the want of necessary accommodations to permit that variety of exercises being made use of, which are requisite to insure complete success. How inferior must such a charity necessarily be, connected with a hospital devoted to other purposes, to one especially set apart for the purpose, be it public or private. Of those of the latter class which I have seen in Europe, that of M. Bouvier has given me the most pleasure. Of the variety and appropriateness of the arrangement to be found at this institution, I shall speak more in detail hereafter.

The couches of extension and segmoid flexion combined with suitable exercises, are the means considered the most effectual, and upon which chief reliance is to be placed, in cases of lateral curvature. As an adjunct to this, and to be used while the patient is walking, &c., these gentlemen make use of some form of spinal support for body apparatus. This consists for the most part of a modification of Tavernier's

Lever Belt, which in a number of cases is without doubt an instrument of great value.

All orthopedic surgeons agree in the necessity of spinal supports being used in some form for spinal curvatures, and for a perfect instrument of this kind, which shall unite the advantages of those we have, without their defects, is what I have sought for diligently. The best which I have seen, where the object has been to go farther than merely to afford support to the spine or staying it in the new position to which other remedies have brought it, until the feeble muscles shall have acquired power to perform their office without this aid, are those employed by Dr. Little, which are still a modification of Tavernier's, but more powerful and better adapted to a great number of cases.

In the treatment of lateral curvature there are of course many other things to be taken into consideration, particularly where there is a scrofulous diathesis, or where the general health is enfeebled. In all cases the attention of the surgeon is to be directed with much care to the less prominent symptoms. Dr. Zinck, of the Orthopedic Institution, Vienna, lays much stress on false positions during sleep, as a cause of this complaint; as, for example, lying with the head inclined toward the left side, thus checking inspiration on that side. He says that on this account the process of cure is rendered much more tedious, and he considers that patients should be watched much during their sleep, that the injury arising from these false positions may be obviated. He has found the muscles of inspiration on the left side in such cases much atrophied.

In the Royal Orthopedic Hospital, London, much reliance is placed upon the instrument to which I have referred above. This was the first orthopedic institution I visited in Europe. It is solely a charitable institution, and owes its origin to the disinterested efforts of Dr. Little, by whom it was carried to a great degree of perfection and usefulness. Mr. Tamplis is now the senior, and Mr. Lonsdale the junior surgeon. The latter gentleman has already made his appearance before the English surgical community, as the inventor of two or three surgical instruments and apparatus. One, in particular, for the fracture of the lower jaw, bids fair to be an instrument of great value.

This Institution or Hospital has accommodations for about forty patients. During the last year seventy-five patients have been admitted, of whom forty-eight have been discharged cured, and eighteen relieved. The number of out-patients is, however, very large. There are two days in the week appointed for their reception, and on these occasions from sixty to eighty patients constantly present themselves. These are for the most part different on different days, and the whole number dependent upon the institution for advice is nine hundred and sixty-nine. The whole number which have been treated at this institution since its formation, is four thousand. It is supported entirely by subscriptions, which now amount to £1917. The list is headed by Prince Albert, followed by a number of the nobility.

The treatment of club feet is a subject which is now so thoroughly understood on both sides of the water, that I need scarcely refer to it here. The chief difference consists in the form of apparatus employed. That of the model which I brought with me from Boston, is considered by Little, Guerin and others, as one of the most perfect they have seen; and, as Dr. L. observed, when speaking of the various apparatus in use, the great expense of this kind of *especial* work in London, is the only reason why this more perfect form cannot be universally adopted.

In some future communication, I shall take occasion to refer to a novel and effectual method now pursued for straightening the bent limbs of rickety children, for the diagnosis and cure of stammering in those cases which admit of a cure, and also for the treatment of some of the varieties of scrofulous diseases and of nervous debility. Until which time, I remain,

Sir, Your most obedient servant,

Buckminster Brown.

